# Successful use of extracorporeal life support and hemadsorption in the context of venlafaxine intoxication requiring cardiopulmonary resuscitation: a case report

**DOI:** 10.1007/s10047-023-01399-8

**Published:** 2023-04-28

**Authors:** Matthias Hoffmann, Samira Akbas, Rahel Kindler, Dominique Bettex

**Affiliations:** 1 Institute of Anesthesiology, University Hospital Zurich, Raemistrasse 100, 8091 Zurich, Switzerland; 2 Institute of Intensive Care Medicine, University Hospital Zurich, Raemistrasse 100, 8091 Zurich, Switzerland

**Keywords:** Cardiopulmonary resuscitation, Venlafaxine intoxication, ECLS, CytoSorb^®^, Takotsubo

## Abstract

Venlafaxine is a serotonin and noradrenalin reuptake inhibitor prescribed as an antidepressant. Overdose clinically manifests with neurological, cardiovascular and gastrointestinal abnormalities based on, amongst others, serotonin syndrome and can be life-threatening due to cardiovascular collapse. Besides immediate decontamination via gastric lavage and inhibition of enteral absorption through active charcoal, successful hemadsorption with CytoSorb^®^ has been reported. We present the case of a 17-year-old female who required extracorporeal life support (ECLS) for cardiovascular collapse as a result of life-threatening venlafaxine intoxication. Serial serum blood concentrations of venlafaxine/desmethylvenlafaxine on admission at a tertiary hospital (approx. 24 h after ingestion) and subsequently 6 h and 18 h thereafter, as well as on days 2 and 4, were measured. CytoSorb^®^ was initiated 6 h after admission and changed three times over 72 h. The initial blood concentration of venlafaxine/desmethylvenlafaxine was 53.52 µmol/l. After 6 h, it declined to 30.7 µmol/l and CytoSorb^®^ was initiated at this point. After 12 h of hemadsorption, the blood level decreased to 9.6 µmol/l. On day 2, it was down to 7.17 µmol/l and decreased further to 3.74 µmol/l. Additional continuous renal replacement therapy using CVVHD was implemented on day 5. The combination of hemadsorption, besides traditional decontamination strategies along maximal organ supportive therapy with ECLS, resulted in the intact neurological survival of the highest venlafaxine intoxication reported in the literature to date. Hemadsorption with CytoSorb^®^ might help to reduce blood serum levels of venlafaxine. Swift clearance of toxic blood levels may support cardiovascular recovery after life-threatening intoxications.

## Background

Venlafaxine is a serotonin, noradrenaline and partial dopamine reuptake inhibitor prescribed as an antidepressant [1]. According to the Swiss poisons information center (Tox Info Suisse), they were approached for toxicological support in venlafaxine intoxications by clinicians approximately 170 times in 2021 and 130 times in 2020 (population of Switzerland approximately 8,700,000). Overdose clinically manifests with neurological, cardiovascular and gastrointestinal abnormalities based on, amongst others, serotonin syndrome (anxiety, disorientation, agitated delirium, clonus, seizure, hyperreflexia, diaphoresis, tremor, hypertension, tachycardia, vomiting, diarrhea). It can be life-threatening due to cardiovascular collapse [2].

## Case report

A 17-year-old female patient was admitted to a regional hospital for mixed intoxication with a presumed intake of 24 g of venlafaxine (both immediate and extended-release preparations) and unknown amounts of oxycodone, zolmitriptan and itinerol B6. The patient had been treated with venlafaxine by her outpatient psychiatrist for severe depression with suicidal ideation for two weeks prior to the event. An inpatient psychiatric stay had already been planned. Approximately five hours after taking the medication, the patient was found somnolent and brought to the hospital by ambulance. Due to the severity of the intoxication with the risk of developing hemodynamic instability, the patient was immediately transferred to the intensive care unit.

**Fig. 1:**
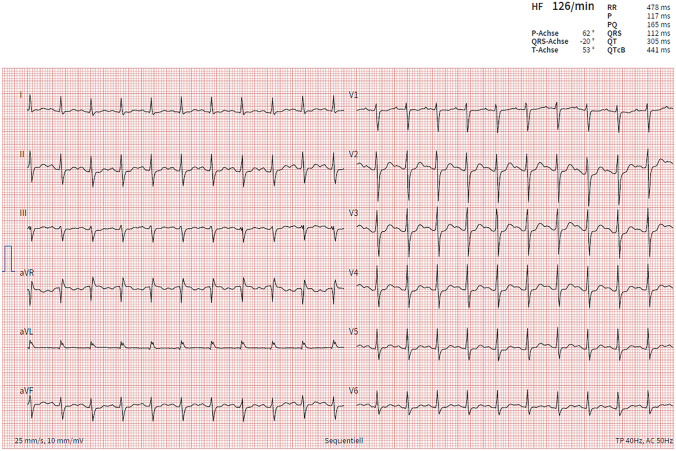
ECG on day of admission with sinus tachycardia and prolonged cQT 441 ms. Running speed 25 mm/s

Shortly after that, recurrent generalized seizures occurred. Due to a status epilepticus, the patient was then analgosedated and intubated. After tracheal intubation, progressive hemodynamic deterioration occurred with sinus tachycardia up to 140 bpm, hypotension with systolic blood pressure of 70 mmHg and centralization (prolonged capillary refill time > 3 s). Figure 1 shows the ECG on the day of admission with sinus tachycardia and a prolongation of the cQT-time. Echocardiography revealed severely impaired left ventricular function (EF 10–15%) with hypokinetic left ventricle, apical and midventricular akinesia with normal right ventricular function. Pericardial effusion was excluded. Arterial blood gas analysis showed metabolic acidosis (pH 7.28) and a serum lactate of 7.8 mmol/l. Despite extended catecholamine therapy with high-dose norepinephrine, dobutamine and adrenaline, the patient could not be stabilized and cardiopulmonary resuscitation due to cardiac arrest was initiated. After 2.5 h (150 min) of mechanical resuscitation, extracorporeal life support (ECLS) system was established on-site with subsequent air-bound transfer to a tertiary hospital.

**Fig. 2:**
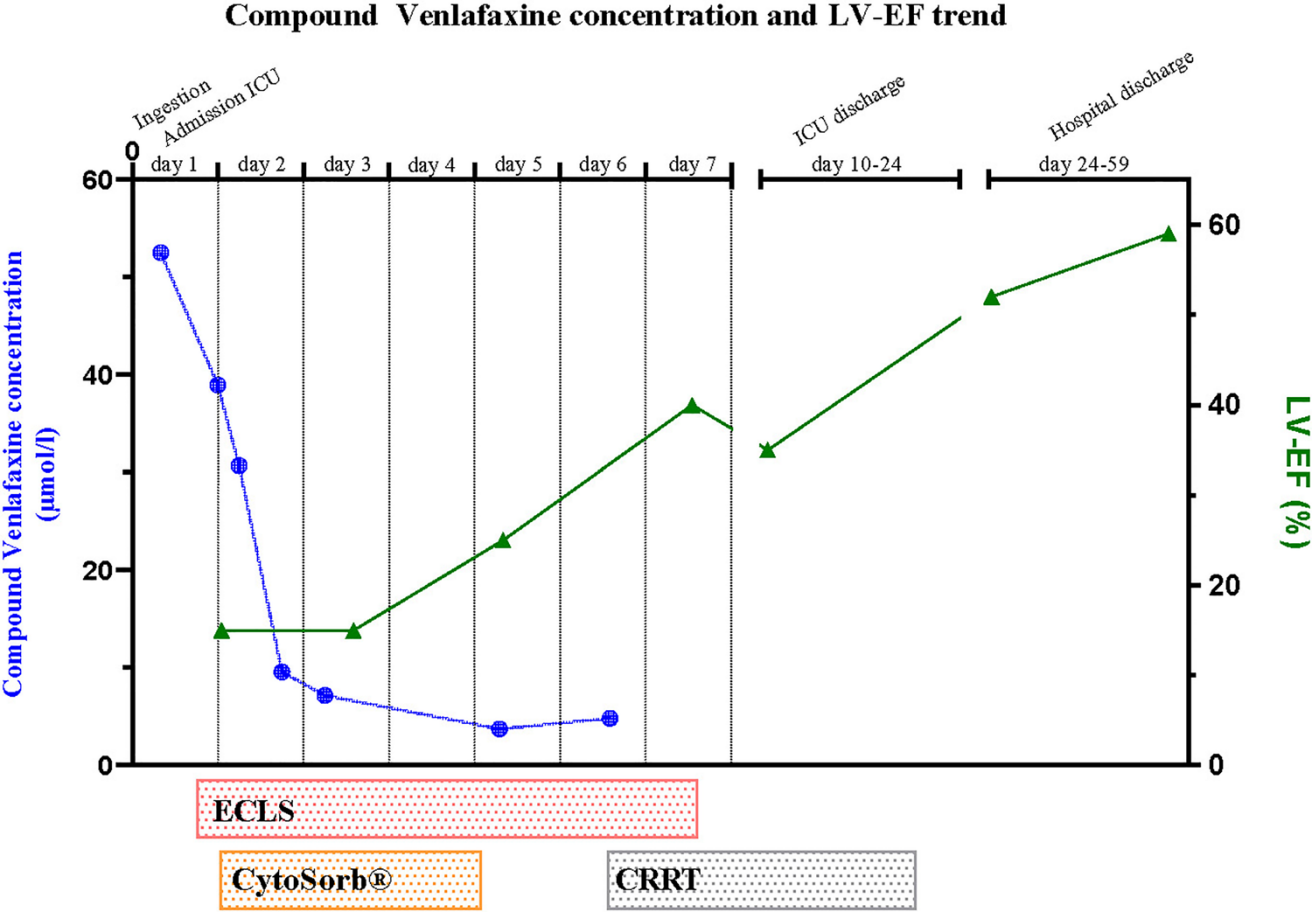
On the left *Y*-axis in blue, plot of the time course of compound plasma concentration of venlafaxine. On the right *Y*-axis in green, time course of left ventricular ejection fraction (LV-EF) in percent. On the upper *X*-axis days since ingestion are displayed. Below the *X*-axis, plot using bars of the duration of use of ECLS, Cytosorb^®^, and hemofiltration. *ECLS* extracorporeal life support, *CRRT* continuous renal replacement therapy

Shortly after arrival at the tertiary hospital, a large volume of tablets (filling one-third of the stomach) was removed during primary decontamination via gastroscopy. The gastric mucosa was slightly hemorrhagic. A total of 55 g of activated charcoal was applied for additional adsorption. Because of a distended abdomen with subileus, repetitive administration was withheld. Laboratory chemistry revealed disseminated intravascular coagulation (DIC) and acute liver failure with transaminase elevation (max. AST 4226 U/l, max. ALT 3224 U/l), drop in coagulation factor V (Factor V: < 10%), INR elevation (max. INR 5.9) and lactic acidosis (Lactate max. 9.8 mmol/l, pH min. 7.27), leading to the administration of N-acetylcysteine following Prescott schema for four days despite negative paracetamol serum levels. Sonographically, the liver was well perfused without obstructive intra- or extrahepatic cholestasis. The patient was anuric with acute kidney injury (AKIN stage 3, max. creatinine 331mcmol/l) and required continuous hemodiafiltration from day five. Toxicological screening in urine and blood detected the metabolites of oxycodone, tramadol, nicotine and lidocaine, venlafaxine, metoprolol, metoclopramide, naloxone, and caffeine. In addition, iatrogenic amoxicillin, midazolam and levetiracetam metabolites were found. The initial compound venlafaxine/desmethylvenlafaxine plasma concentration was markedly elevated (maximum 52.53 µmol/l) but reduced significantly (9.60 µmol/l) within the first 24 h under initiated ECLS therapy with CytoSorb^®^ filter. The adsorption filter (CytoSorb^®^, blood flow 300 ml/min) was changed three times over 72 h and removed after three days of therapy as recommended by the manufacturer. Figure 2 shows the course of venlafaxine/desmethylvenlafaxine plasma concentration and LV-EF (left ventricular ejection fraction) graphically. Balanced hemodynamic management using volume and low-dose epinephrine (0.1 µg/kg/min) to promote inotropy, as well as high ECLS blood flow (maximum 5 l/min), were used to maintain sufficient mean arterial pressure. A 900 ml serous left pleural effusion was drained following the correction of coagulation on day four. Already at admission, microbiological sampling was performed after documented aspiration and the established antimicrobial therapy with amoxicillin/clavulanic acid was continued. Despite negative bacterial detection, antimicrobial therapy was escalated to piperacillin/tazobactam on day eight due to respiratory deterioration and increasing inflammatory parameters (CRP peak level 154 mg/l).

The patient’s health condition progressively improved over the next few days. In addition to an increased blood pressure amplitude over 20 mmHg, serial transthoracic echocardiograms (TTE) documented improved cardiac function and sufficient ejection fraction (EF) of approximately 35% under ECLS blood flow of 3–4 l. Electrocardiographically, cQT peaked at 507 ms with no arrhythmias. Three days post-admission, levosimendan (0.1mcg/kg/min) perfusion (25 mg) was followed by weaning and removal of the ECLS system. Hepatic function recovered and after 7 days of high-volume hemodiafiltration, acid–base and fluid hemostasis were restored. The patient was transferred back to the peripheral hospital on day 11 after symptom onset and completely recovered there neurologically and cardiopulmonarily. The discharge to inpatient psychiatric treatment was organized 31 days after intoxication due to persistent suicidality.

## Discussion

This case report describes a cardiogenic shock with prolonged resuscitation due to severe serotonergic syndrome and takotsubo-like cardiac failure in the context of intoxication with 24 g of venlafaxine, the highest proven survived dose to date. With a maximum serum concentration of 52.53 µmol/l, the patient exceeded the therapeutic range of 0.7–1.44 µmol/l by a factor of 36 [3]. Venlafaxine intoxication, in addition to neurologic symptoms, leads to left-leading cardiac insufficiency, which may mimic a takotsubo cardiomyopathy and is life-threatening [4, 5]. The pathophysiological processes of these intoxication symptoms are not fully understood [2]. One hypothesis is myocardial stunning caused by excessive adrenergic stimulation, similar to the possible etiology of takotsubo cardiomyopathy [5, 6]. Alternatively, depending on the venlafaxine concentration, there is a decrease in inward sodium current and thus inhibition of the action potential, which also results in myocardial dysfunction [7, 8]. In line with this, our patient’s electrocardiogram (ECG) showed cQT prolongation up to 507 ms without relevant arrhythmias. Since no antidote is available to date, intoxication with venlafaxine is treated supportively. In particularly severe cases, the use of ECLS, some of them in combination with CytoSorb^®^, has been shown to be successful and may improve survival in poisoned patients experiencing severe shock [9–13].

CytoSorb^®^ is an adsorption device that primarily adsorbs cytokines and other molecules, including bilirubin or drugs with the size of 5–55 kDa along the concentration gradient [14]. The cartridge of the CytoSorb^®^ device consists of coated polystyrene divinyl benzene copolymer beads, which have a size of 300–800 ym and pores of 20–50 A. The device is successfully used in septic patients or in the context of post-resuscitation syndrome [15]. In in vitro studies, CytoSorb^®^ was also able to absorb different drugs, which differed in molecular weight, protein binding and solubility [14]. To date, only case reports exist on the use of CytoSorb^®^ in ECLS therapy for venlafaxine or other drug intoxications and the application remains off-label [10, 16, 17]. In order to possibly reduce the serum concentration and thus the toxicity of venlafaxine, we used the hemadsorption filter CytoSorb^®^ in addition to the established ECLS. The half-life of 15 h, which is prolonged in venlafaxine intoxication due to its change in elimination kinetics, could be reduced under the combination of these therapy elements, as the plasma concentration dropped from 30.70 µmol/l to 9.60 µmol/l within 12 h [18]. At this time, the patient was not yet on hemodialysis, so a contributing effect of continuous renal replacement therapy can be excluded. We therefore postulate that the use of ECLS in combination with a hemadsorption filter, such as CytoSorb^®^, might has had an effect on the detoxification process. Because of the aforementioned risk of agglutination with possible mechanical problems and lipid deposition in the ECLS system and CytoSorb^®^, we decided against applying intravenous lipid emulsion for hemodynamic instability. However, its use is successfully described in a case report by Schroeder et al. [10, 19].

## Conclusion

We present the case report of a patient with prolonged resuscitation requirement and subsequent ECLS who survived the highest described venlafaxine ingestion. Using ECLS in combination with the hemadsorption filter CytoSorb^®^ resulted in patient survival with good neurological outcome.

Although the impact of the CytoSorb^®^ filter on patient outcome is not specifically measurable in our conducted laboratory analysis, it nevertheless shows a congruent course to previous clinical cases described in other case reports. This could support potential use of a hemadsorption filter in the context of intoxication with venlafaxine.

## Data Availability

The datasets used and/or analyzed during the current study are available from the corresponding author on reasonable request.

## Abbreviations

DIC: Disseminated intravascular coagulation
ECG: Electrocardiogram
ECLS: Extracorporeal life support
ECMO: Extracorporeal membrane oxygenation
TTE: Transthoracic echocardiography
